# The “Grep” Command But Not FusionMap, FusionFinder or ChimeraScan Captures the *CIC-DUX4* Fusion Gene from Whole Transcriptome Sequencing Data on a Small Round Cell Tumor with t(4;19)(q35;q13)

**DOI:** 10.1371/journal.pone.0099439

**Published:** 2014-06-20

**Authors:** Ioannis Panagopoulos, Ludmila Gorunova, Bodil Bjerkehagen, Sverre Heim

**Affiliations:** 1 Section for Cancer Cytogenetics, Institute for Cancer Genetics and Informatics, The Norwegian Radium Hospital, Oslo University Hospital, Oslo, Norway; 2 Centre for Cancer Biomedicine, Faculty of Medicine, University of Oslo, Oslo, Norway; 3 Department of Pathology, The Norwegian Radium Hospital, Oslo University Hospital, Oslo, Norway; 4 Faculty of Medicine, University of Oslo, Oslo, Norway; European Institute of Oncology, Italy

## Abstract

Whole transcriptome sequencing was used to study a small round cell tumor in which a t(4;19)(q35;q13) was part of the complex karyotype but where the initial reverse transcriptase PCR (RT-PCR) examination did not detect a *CIC-DUX4* fusion transcript previously described as the crucial gene-level outcome of this specific translocation. The RNA sequencing data were analysed using the FusionMap, FusionFinder, and ChimeraScan programs which are specifically designed to identify fusion genes. FusionMap, FusionFinder, and ChimeraScan identified 1017, 102, and 101 fusion transcripts, respectively, but *CIC-DUX4* was not among them. Since the RNA sequencing data are in the fastq text-based format, we searched the files using the “grep” command-line utility. The “grep” command searches the text for specific expressions and displays, by default, the lines where matches occur. The “specific expression” was a sequence of 20 nucleotides from the coding part of the last exon 20 of *CIC* (Reference Sequence: NM_015125.3) chosen since all the so far reported *CIC* breakpoints have occurred here. Fifteen chimeric *CIC-DUX4* cDNA sequences were captured and the fusion between the *CIC* and *DUX4* genes was mapped precisely. New primer combinations were constructed based on these findings and were used together with a polymerase suitable for amplification of GC-rich DNA templates to amplify *CIC-DUX4* cDNA fragments which had the same fusion point found with “grep”. In conclusion, FusionMap, FusionFinder, and ChimeraScan generated a plethora of fusion transcripts but did not detect the biologically important *CIC-DUX4* chimeric transcript; they are generally useful but evidently suffer from imperfect both sensitivity and specificity. The “grep” command is an excellent tool to capture chimeric transcripts from RNA sequencing data when the pathological and/or cytogenetic information strongly indicates the presence of a specific fusion gene.

## Introduction

The translocation t(4;19)(q35;q13) was described by Richkind et al [1] as the sole chromosomal aberration in a tumor diagnosed as poorly differentiated extraskeletal mesenchymal sarcoma in a 12-year-old-boy. The authors mentioned that a similar translocation had also been reported as part of complex karyotype in an embryonal rhabdomyosarcoma (RMS) cell line [2] and as part of a three-way translocation t(4;19;12)(q35;q13.1;q13) in an undifferentiated/embryonal RMS [3] and suggested that it might be a recurrent chromosomal aberration in malignant primitive mesenchymal stem cells [1]. Sommers et al [4] described a subcutaneous primitive neuroectodermal tumor/Ewing sarcoma without *EWSR1* rearrangement but with a complex karyotype containing a t(4;19)(q33∼35;q13). Kawamura-Saito et al [5] described two cases of Ewing-like sarcoma which had a t(4;19)(q35;q13) in their karyotypes. They also showed that the translocation resulted in fusion of the capicua transcriptional repressor *CIC* gene on 19q13, which codes for a high mobility group box transcription factor, with the double homeodomain *DUX4* gene on 4q35 [5].

*DUX4* is located within a D4Z4 repeat array in the subtelomeric region of chromosome arm 4q [6]. A similar D4Z4 repeat array has been identified on chromosome 10 [7]. Each D4Z4 repeat unit has an open reading frame (named *DUX4*) that encodes two homeoboxes [6]. There is no evidence for transcription of this gene from standard cDNA libraries, but RT-PCR and in vitro expression experiments indicate that the ORF is transcribed [8], [9]. The encoded protein is located in the nucleus, induces cell death, and has been reported to function as a transcriptional activator of paired-like homeodomain transcription factor 1 (PITX1) [8], [9]. So far, there are roughly 20 reported cases of sarcoma with the t(4;19)(q35;q13) and/or *CIC-DUX4* fusion [1]–[5], [10]–[15]. In seven other cases with *CIC-DUX4*, the *DUX4* gene involved in the fusion apparently stems from the locus on 10q26 [13], [16]. The current data suggest that the *CIC-DUX4* fusion defines a subgroup of primitive round cell sarcomas, different from Ewing sarcoma, with distinctive histopathology and rapid disease progression [1]–[5], [10]–[15].

Recently, whole transcriptome sequencing (RNA-Seq, RNA sequencing) was shown to be an efficient tool in the detection of fusion genes in cancer [17]. In short, extracted RNA from cancer cells is massively sequenced, and then the raw data are analyzed with one or more programs specifically dedicated to the task of detecting fusion transcripts such as ChimeraScan [18], FusionMap [19], and FusionFinder [20]. However, the programs typically identify numerous fusion transcripts making the assessment of which of them are important and which are noise extremely difficult. To overcome this challenge, we and others have used combinations of cytogenetics and RNA-Seq to detect the “primary” fusion genes of neoplasms carrying only one or a few chromosomal rearrangements. A number of fusion genes were found using this approach, among them the recurrent *ZC3H7-BCOR* in endometrial stromal sarcomas [21], *IRF2BP2-CDX1* in a mesenchymal chondrosarcoma [22], and *EWSR1-YY1* in a subset of mesotheliomas [23]. In the present study, we performed whole transcriptome sequencing to study a small round cell tumor in which t(4;19)(q35;q13) was part of a complex karyotype. While the fusion gene detection programs ChimeraScan [18], FusionMap [19], and FusionFinder [20] failed to detected the *CIC-DUX4* fusion transcript, the “grep” command-line utility captured the cytogenetically indicated *CIC-DUX4* fusion gene.

## Materials and Methods

### Ethics Statement

The study was approved by the regional ethics committee (Regional komité for medisinsk forskningsetikk Sør-Øst, Norge, http://helseforskning.etikkom.no). Written informed consent was obtained from the patient prior to her death. The ethics committee approval included a review of the consent procedure and all patient information has been anonymized and de-identified.

### Patient

**Figure 1:**
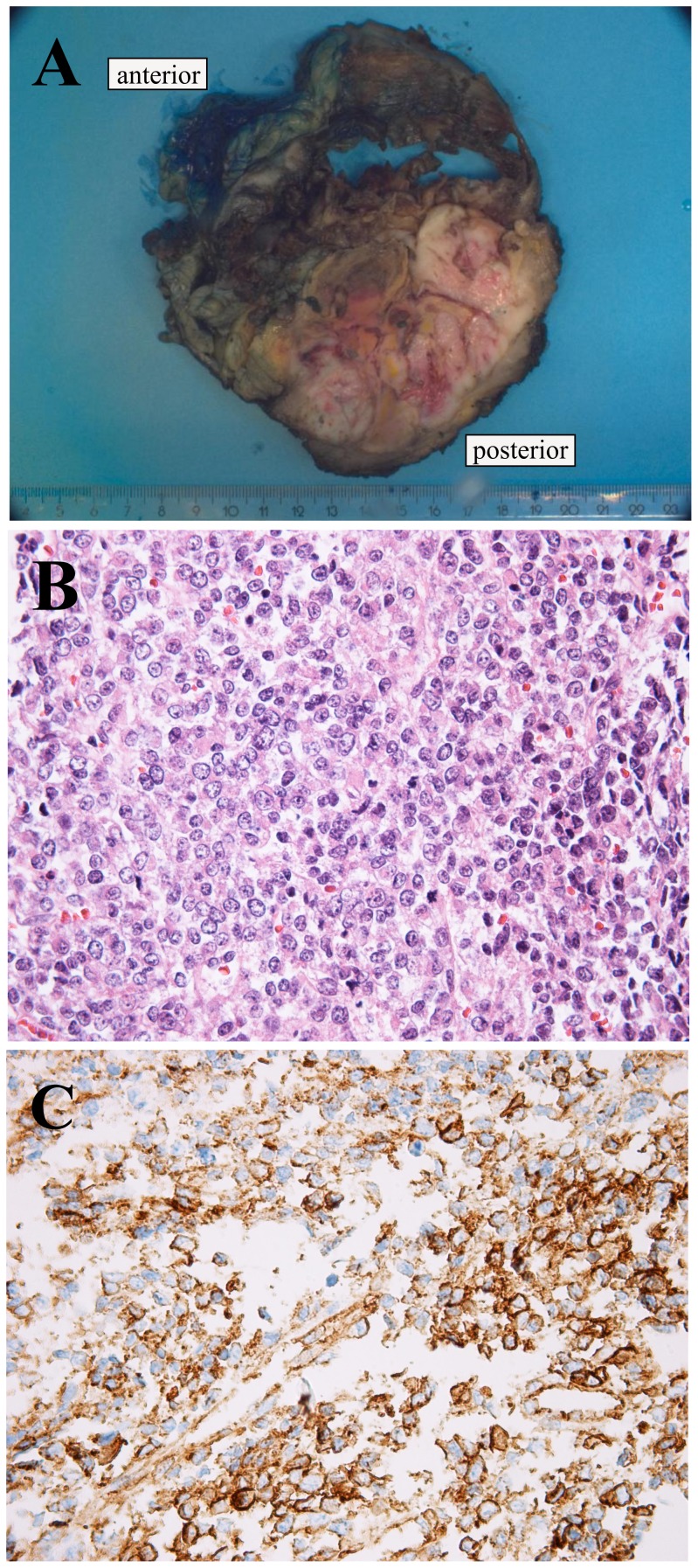
Pathologic examination of the tumor. A) The 12 cm large tumour was localized in the skeletal muscle in the thoracic wall with extension to the retroperitoneum and costae. B) HE-stained slides showed a small round cell tumour. C) Immunexpression of CD99.

A 40-year-old female presented with pain in the lower part of the thoracic wall and imaging showed a tumor in thoracic skeletal muscle with extension into the retroperitoneum and costae. The histological diagnosis was small round cell sarcoma (Figure 1). Immunohistochemistry demonstrated positive findings for vimentin, AE1/AE3, and CD99, but was negative for WT1, CD56, synaptophysin, chromogranin, MYF4, SMA desmin, CD3, CD20, CD45, CD79a, TdT, S100, and FLI1. RT-PCR did not show gene fusion consistent with Ewing sarcoma (*EWSR1-ERG/FLI1*) or synovial sarcoma (*SS18-SSX1*, *2* or *4*). The patient received preoperative chemotherapy and the resected specimen disclosed a 12 cm large tumor. The patient later developed lung metastasis and a local recurrence and died of sarcoma 10 months after the diagnosis.

### Chromosome banding analysis and fluorescence in situ hybridization (FISH)

A sample from the surgically removed tumor was mechanically and enzymatically disaggregated and short-term cultured as described elsewhere [24]. The cultures were harvested and the chromosomes G-banded using Wright stain. The subsequent cytogenetic analysis and karyotype description followed the recommendations of the ISCN [25].

The BAC clone RP11-556K23 (chr19:47422736–47630224), which maps to 19q13.2 and contains the *CIC* gene, was retrieved from the Human genome high-resolution BAC re-arrayed clone set (the “32k set”; BACPAC Resources, http://bacpac.chori.org/pHumanMinSet.htm). Mapping data for the 32k human re-array are available in an interactive web format (http://bacpac.chori.org/pHumanMinSet.htm, from the genomic rearrays page) and can be obtained by activation of the ucsc browser track for the hg17 UCSC assembly from the “32k set” homepage (http://bacpac.chori.org/genomicRearrays.php). FISH mapping of the clone was performed on normal controls to confirm their chromosomal location. DNA was extracted and probes were labelled and hybridized according to Abbott Molecular recommendations (http://www.abbottmolecular.com/home.html). Chromosome preparations were counterstained with 0.2 µg/ml DAPI and overlaid with a 24×50 mm^2^ coverslip. Fluorescent signals were captured and analyzed using the CytoVision system (Leica Biosystems, Newcastle, UK).

### High-throughput paired-end RNA-sequencing

Tumor tissue adjacent to that used for cytogenetic analysis and histologic examination had been frozen and stored at −80°C. Total RNA was extracted from the tumor using Trizol reagent according to the manufacturer's instructions (Invitrogen, Oslo, Norway) and its quality was checked by Experion Automated Electrophoresis System (Bio-Rad Laboratories, Oslo, Norway). Three µg of total RNA from the primary tumor were sent for high-throughput paired-end RNA-sequencing at the Genomics Core Facility, The Norwegian Radium Hospital (http://genomics.no/oslo/). The RNA was sequenced using an Illumina HiSeq 2500 instrument and the Illumina software pipeline was used to process image data into raw sequencing data. Only sequence reads marked as “passed filtering” were used in the downstream data analysis. A total of 100 million reads were obtained. The softwares FusionMap (http://www.omicsoft.com/fusionmap/) [19], Fusion Finder (http://bioinformatics.childhealthresearch.org.au/software/fusionfinder/) [20], and ChimeraScan (https://code.google.com/p/chimerascan/) [18] were used for the discovery of fusion transcripts. In addition, the “grep” command (http://en.wikipedia.org/wiki/Grep) was used to search the fastq files of the sequence data (http://en.wikipedia.org/wiki/FASTQ_format) for *CIC* sequence (NM_015125 version 3).

FusionMap was run on a PCR with Windows XP professional as the operative system. FusionFinder, ChimeraScan, and “grep” command were run on a PC with Bio-Linux 7 as the operating system [26].

### PCR

The primers used for PCR amplification and sequencing are listed in Table 1.

**Table 1:** Primers used for PCR amplifications and sequencing.

Oligo Name	Sequence (5′→3′)
CIC-4105F	CGAAGAGCGCTTTGCTGAGTTGCC
CIC-4283F	AGAAGACGCTCCAGCTGCAGCTCG
CIC-4377F	CCGAGGACGTGCTTGGGGAGCTA
CIC-4453F	GGCCCTGGTCATGCAGCTCTTTCA
CIC-4856R	CTCAGGGGTCCCTCACCTGCCTGT
CIC-4958R	CCCAAACTGGAGAGGACGAAATGGC
DUX4-1053R	ACCGAGGAGCCTGAGGGTGGGAG
DUX4-1151R	CTTGAGCGGGCCCAGGCTGTG
DUX4-1507R	CTTCCAGCGAGGCGGCCTCTTC
DUX4-1538R	GCAGAGCCCGGTATTCTTCCTCGC

One µg of tumor total RNA was reverse-transcribed in a 20 µL reaction volume using iScript Advanced cDNA Synthesis Kit for RT-qPCR according to the manufacturer's instructions (Bio-Rad Laboratories, Oslo, Norway). Initially, the 25 µL PCR-volume contained 12.5 µL of Premix Taq (Takara Bio Europe/SAS, Saint-Germain-en-Laye, France), 1 µL of the synthesized cDNA, and 0.4 µM of each of the forward CIC-4105F and reverse DUX4-1538R primers. One µL of the 1^st^ PCR amplification was used as template in a nested PCR with the forward CIC-4283F and reverse DUX4-1507R primers. For the quality of the cDNA synthesis the primers CIC-4238F and CIC-4958R were used to amplify a CIC cDNA fragment. The PCRs were run on a C-1000 Thermal cycler (Bio-Rad Laboratories) with the following cycling conditions: an initial denaturation at 94°C for 30 sec followed by 35 cycles of 7 sec at 98°C and 2 min at 68°C, and a final extension for 5 min at 68°C.

In subsequent PCR amplifications, PrimeSTAR GXL DNA polymerase was used (Takara Bio). According to the company's information this is a high fidelity polymerase suitable for GC-rich templates that are otherwise difficult to amplify. The 25 µL PCR volume contained 1× PrimeSTAR GXL Buffer (Takara Bio), 1 µL of the synthesized cDNA, 200 µM of each dNTP, 0.4 µM of each of the forward primer CIC-4377F and the reverse primer DUX4-1151R or 0.4 µM of each of the primers CIC-4453F and DUX4-1053R. The PCR was run on a C-1000 Thermal cycler (Bio-Rad Laboratories) with an initial denaturation at 94°C for 30 sec, followed by 35 cycles of 7 sec at 98°C, 2 min at 68°C, and a final extension for 5 min at 68°C. Three µL of the PCR products were stained with GelRed (Biotium, Hayward, CA, USA), analyzed by electrophoresis through 1.0% agarose gel, and photographed.

The rest of the amplified PCR products were purified using the NucleoSpin Gel and PCR Clean-up kit (Macherey-Nagel, VWR International, Oslo, Norway). Direct sequencing (Sanger sequencing) was performed using the light run sequencing service of GATC Biotech (http://www.gatc-biotech.com/en/sanger-services/lightrun-sequencing.html). The BLAST software (http://blast.ncbi.nlm.nih.gov/Blast.cgi) was used for computer analysis of sequence data. The nucleotide sequence has been deposited in the GenBank with accession number KJ670706.

## Results

**Figure 2:**
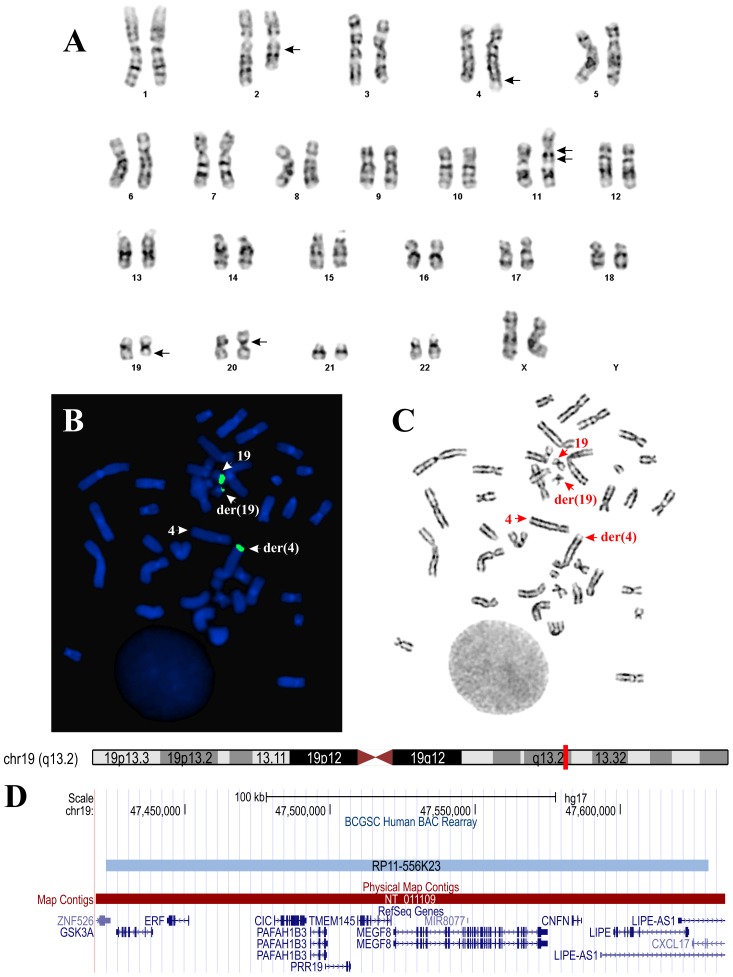
Cytogenetic and FISH analyses of the tumor. A) Karyogram showing chromosome aberrations del(2)(q13q23), t(4;19)(q35;q13), ins(11;?)(q11;?), and der(20)?t(20;20)(p11;q11); breakpoints are indicated by arrows. B) FISH performed on metaphase spread using BAC RP556K23 (green signal) from 19q13 containing the CIC gene. A part from this probe has moved to the derivative chromosome 4. The der(4), der(19), and the normal chromosomes 4 and 19 are indicated by arrows. C) G- banding of the metaphase spread shown in (B). The der(4), der(19) and the normal chromosomes 4 and 19 are indicated by arrows. D) The location of the BAC RP556K23 on chromosome 19 and the genes found in this region. The data obtained from UCSC Genome Browser (http://genome.ucsc.edu/).

G-banding analysis yielded the diagnostic karyotype 46,XX,del(2)(q13q23),t(4;19)(q35;q13),ins(11;?)(q11;?),der(20)?t(20;20)(p11;q11)[14]/46,XX[3] (Figure 2A). When metaphase spreads (Figure 2B) were hybridized with the BAC- RP11-556K23, one split signal was seen, indicating that the translocation breakpoint on chromosome 19 was within the BAC (Figure 2C). This clone contains, apart from *CIC*, the genes *GSK3A*, *ERF*, *PAFAH1B3*, *PRR19*, *TMEM145*, *MEGF8*, *CNFN*, and *LIPE* (Figure 2D).

**Figure 3:**
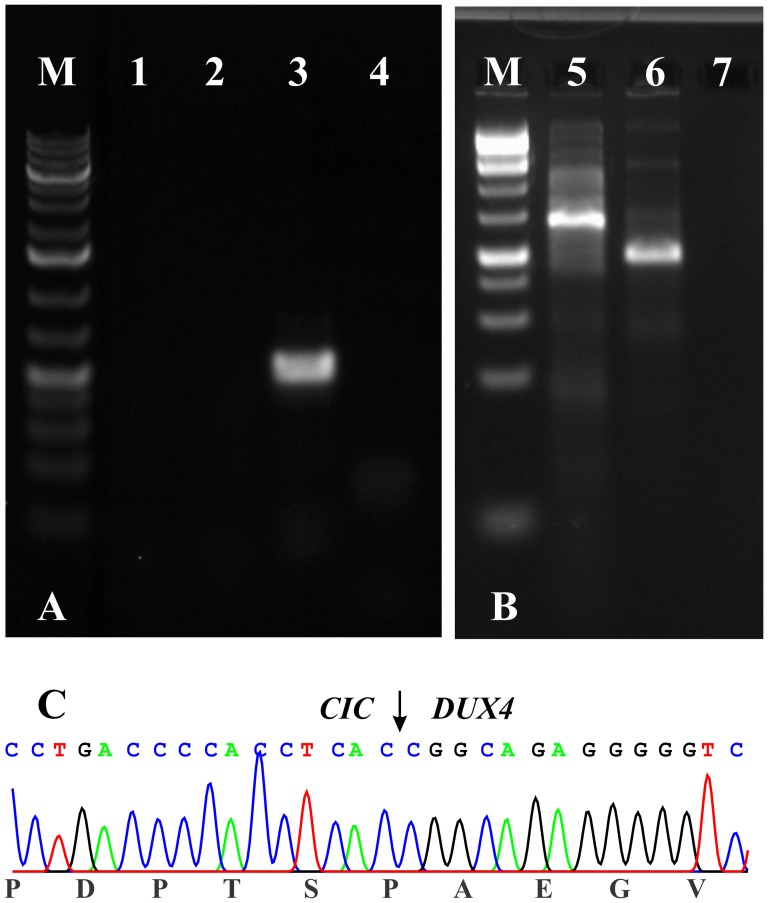
RT-PCR results for the expression of CIC-DUX4 in the tumor. A) The initial PCR with Premix Taq and the primer set CIC-4105F/DUX4-1538R (lane 1) as well as the nested PCR with the primers CIC-4283F/DUX4-1507R (lane 2) did not amplify any cDNA fragments. The primer set CIC-4238F/CIC-4958R (lane 3) amplified a CIC cDNA fragment suggesting the good quality of the synthesized cDNA. Lane 4, Blank, no RNA in cDNA synthesis. B) PCR amplifications using the PrimeSTAR GXL DNA polymerase and the primer combinations CIC-4377F/DUX4-1151R (lane 5) and CIC-4453F/DUX4-1053R (lane 6). Lane 7, Blank, no RNA in cDNA synthesis. M, 1 Kb DNA ladder (GeneRuler, Fermentas). C) Partial sequence chromatogram of the amplified cDNA fragment showing that CIC is fused to DUX4.

The initial PCR with Premix Taq and the primer set CIC-4105F/DUX4-1538R as well as the nested PCR with the primers CIC-4283F/DUX4-1507R failed to amplify any cDNA fragments. However, the primer set CIC-4238F/CIC-4958R amplified a *CIC* cDNA fragment suggesting that the synthesized cDNA was of good quality (Figure 3A). Because of the negative RT-PCR results, whole transcriptome sequencing was performed and the sequencing data were analyzed with FusionMap, FusionFinder, and ChimeraScan which are programs designed to detect fusion genes from high throughput sequencing data [18], [19], [20].

FusionMap identified 1024 potential fusion transcripts (Table S1) but *CIC-DUX4* was not among them. Neither *GSK3A*, *ERF*, *PAFAH1B3*, *PRR19*, *TMEM145*, *MEGF8*, *CNFN*, nor *LIPE*, the other genes which are localized on the FISH probe, were found to be partners in the detected fusion transcripts. FusionFinder and ChimeraScan identified 103 and 101 potential fusion transcripts, respectively (Tables S2 and S3), but again *CIC-DUX4* was not among them. Neither *GSK3A*, *ERF*, *PAFAH1B3*, *PRR19*, *TMEM145*, *MEGF8*, *CNFN* nor *LIPE*, the other genes within the BAC, were found to be partners in the detected fusion transcripts.

Since fastq is a text-based format of the sequence data, we decided to use the “grep” command-line utility and search for sequences which contained part of the last exon of *CIC* (exon 20, nucleotides 4500–5473 in the sequence with accession number NM_015125 version 3). The search terms were “GCCGCCTTCCAGGCCCGCTA” (nt 4511–4530) and “CAGGGGGCCCTGACCCCACC” (nt 4701–4720). The first search term extracted 76 sequences containing *CIC* cDNA fragments (data not shown). The second search term extracted 22 sequences. Blasting of each of these sequences with the human genomic plus transcript database (http://blast.ncbi.nlm.nih.gov/Blast.cgi), *CIC* mRNA reference sequence NM_015125.3, and *DUX4* mRNA reference sequence NM_033178.4 showed that 15 out of the 22 were chimeric *CIC-DUX4* cDNA fragments (Table 2). The fusion had occurred between nt 4724 of *CIC* mRNA reference sequence NM_015125.3 and nt 771 of *DUX4* mRNA reference sequence NM_033178.4. Using the search term “CCCACCTCACCGGCAGAGGG” which is composed of 10 nt of *CIC* (CCCACCTCAC) and 10 nt (CGGCAGAGGG) of *DUX4* upstream and downstream of the fusion point, 19 sequences were retrieved, 15 of which were those found with the “CAGGGGGCCCTGACCCCACC” search term.

**Table 2:** The 22 sequences with the “grep” command line utility with the search term “CAGGGGGCCCTGACCCCACC” (in bold italics).

**Line**	**Sequences**
1	CCCACTCCCAGCCCCG***CAGGGGGCCCTGACCCCACC***TCACCCAGCTCGGACTCTGGCACGGCCCAGGCTGCCCCGCCACTGCCTCCACCCCCCGAGTCGGG
2	CACTCCCAGCCCCG***CAGGGGGCCCTGACCCCACC***TCAC**cggcagagggggtctcccaacctgccccggcgcgcggggatttcgcctacgccgccccggctc**
3	CGCCCCCCACTGGCACCGCTGCTGCCCCTGCCCCCACTCCCAGCCCCG***CAGGGGGCCCTGACCCCACC***TCAC**cggcagaggggggctcccaacctgccccg**
4	CGCTGCTGCCCCTGCCCCCACTCCCAGCCCCG***CAGGGGGCCCTGACCCCACC***TCAC**cggcagagggggtctcccaacctgccccggcgcgcggggatttcg**
5	CCTGCCCCCACTCCCAGCCCCG***CAGGGGGCCCTGACCCCACC***TCAC**cggcagagggggtctcccaacctgccccggcgcgcggggatttcgcctacgccgc**
6	CACCGCTGCTGCCCCTGCCCCCACTCCCAGCCCCC***CAGGGGGCCCTGACCCCACC***TCAC**cggcagagggggcctcccaacctgccccggcgcgcggggatt**
7	TGGCACCGCTTCTGCCCCTGCCCCCACTCCCAGCCCCG***CAGGGGGCCCTGACCCCACC***TCACCCAGCTCGGACTCTGGCACGGCCCAGGCTGCCCCGCCAC
8	CCTGCCCCCACTCCCAGCCCCG***CAGGGGGCCCTGACCCCACC***TCAC**cggcagagggggtctcccaacctgccccggcgcgcggggatttcgcctacgccgc**
9	CGCTGCTGCCCCTGCCCCCACTCCCAGCCCCG***CAGGGGGCCCTGACCCCACC***TCACTTGCAGAGGGGGTCTCCCAACCTGCCCCGGCGCGCGAGAGATCGG
10	CCTCTCCCTGTACCGCCCCCCACTGGCACCGCTGCTGCCCCTGCCCCCACTCCCAGCCCCG***CAGGGGGCCCTGACCCCACC***TCAC**cggcagagggggtctc**
11	CCCAGCCCCG***CAGGGGGCCCTGACCCCACC***TCACCCAGCTCGGACTCTGGCACGGCCCAGGCTGCCCCGCCACTGCCTCCACCCCCCGAGTCGGGGCCTGG
12	CCTGCCCCCACTCCCAGCCCCG***CAGGGGGCCCTGACCCCACC***TCAC**cggcagagggggactcccaacctgccccggcgcgcggggatttcgcctacgccgc**
13	CCCCACTCCCAGCCCCG***CAGGGGGCCCTGACCCCACC***TCAC**cggcagagggggtctcccaacctgccccggcgcgcggggatttcgcctacgccgccccgg**
14	CTCCCAGCCCCG***CAGGGGGCCCTGACCCCACC***TCAC**cggcagagggggtctcccaacctgccccggcgcgcggggatttcgcctacgccgccccggctcct**
15	CCACTGCCAGCCCCG***CAGGGGGCCCTGACCCCACC***TCACCCAGCTCGGACTCTGGCACGGCCCAGGCTGCCCCGCCACTGCCTCCACCCCCCGAGTCGGGG
16	CACTCCCAGCCCCG***CAGGGGGCCCTGACCCCACC***TCAC**cggcagagggggtctcccaacctgccccggcgcgcgggggtttcgcctacgccgccccggctc**
17	CCCCTGCCCCCACTCCCAGCCCCG***CAGGGGGCCCTGACCCCACC***TCAC**cggcagagggggcctcccaacccgcccccgcgcgcgggggctgcgcctacgcc**
18	CCCACTCCCAGCCCCG***CAGGGGGCCCTGACCCCACC***TCAC**cggcagagggggtctgccaacctgccccggcgcgcggggatttcgcctacgccgccccggc**
19	CCGCTGCTGCCCCTGCCCCCACTCCCAGCCCCG***CAGGGGGCCCTGACCCCACC***TCACCCAGCTCGGACTCTGGCACGGCCCAGGCTGCCCCGCCACTGCCC
20	CACCGCTGCTGCCCCTGCCCCCACTCCCAGCCCCG***CAGGGGGCCCTGACCCCACC***TCACCGGCAGTGGGGGTCTCCCAACCTGCCCCGGCGCGCGGGGATT
21	GGCACCGCTGCTGCCCCTGCCCCCACTCCCAGCCCCG***CAGGGGGCCCTGACCCCACC***TCAC**cggcagagggggtctcccaacctgccccggcgcgcggggg**
22	CCAGCCCCG***CAGGGGGCCCTGACCCCACC***TCAC**cggcagagggggtctcccaacctgccccggcgcgcggggatttcgcctacgccgccccggctcctccc**

The CIC sequences are shown in uppercase letters. DUX4 sequences are shown in bold lowercase letters.

To verify the data obtained with the “grep” command, PCR amplifications were performed using the PrimeSTAR GXL DNA polymerase. Both primer combinations, CIC-4377F/DUX4-1151R and CIC-4453F/DUX4-1053R, amplified cDNA fragments (Figure 3B). Sanger sequencing verified that they were *CIC-DUX4* fusion transcripts which had the same fusion point found with the “grep” command (Figure 3C).

## Discussion

Our initial negative result for *CIC-DUX4* fusion with RT-PCR prompted us to investigate the tumor using whole transcriptome sequencing. The small round cell tumor had the t(4;19)(q35;q13) translocation as part of its karyotype and in addition a split signal of the BAC RP11-556K23 (mapped on 19q13), which contains *CIC*, features that led us to nevertheless believe strongly that a *CIC-DUX4* fusion must be present. However, also *GSK3A*, *ERF*, *PAFAH1B3*, *PRR19*, *TMEM145*, *MEGF8*, *CNFN*, and *LIPE* were present in the BAC bridging the breakpoint and could conceivably be the gene-level target of the chromosomal split. It was therefore surprising that no signs of any *CIC-DUX4* were evident when we analyzed the raw sequencing data using ChimeraScan [18], FusionMap [19], and FusionFinder [20], fusion-finder programs that have all been evaluated recently on a synthetic dataset as well as real datasets that included experimentally validated chimeras [27], [28]. All three programs produced a plethora of fusion transcripts but none of them contained *CIC* or any of the other 8 genes found in the split RP11-556K23 FISH probe. We then as a last resort decided to search for *CIC* sequences in the whole transcriptome sequencing data set using the “grep” command-line utility. The rationale was: 1) the RNA sequencing data are in fastq format files (filename.fastq) and fastq is a text-based format (http://en.wikipedia.org/wiki/FASTQ_format) and 2) the sequence data can be searched using the “grep” command-line utility (http://en.wikipedia.org/wiki/Grep). The “grep” command-line utility is used for searching text or a file for specific expressions. By default, “grep” displays the lines where matches occur. Our “specific expression” was a sequence of 20 nucleotides from the coding part of the last exon (20) of *CIC* (Reference Sequence: NM_015125.3) since all the so far reported *CIC* breakpoints have occurred in that part of the *CIC* gene [5], [12]–[14]. The sequences obtained by “grep” were blasted against the human genomic plus transcript database (http://blast.ncbi.nlm.nih.gov/Blast.cgi) in order to identify possible chimeric fragments containing part of *CIC* and part of another gene.

This approach allowed us to obtain from the RNA sequencing fastq file 15 chimeric *CIC-DUX4* cDNA sequences (Table 2) and to map the fusion between the *CIC* and *DUX4* genes precisely. Subsequently, four more chimeric *CIC-DUX4* sequences were identified using a 20-mer sequence containing the fusion point as “specific expression” in the “grep” command-line utility. The fusion occurred between nt 4724 of *CIC* mRNA reference sequence NM_015125.3 and nt 771 of *DUX4* mRNA reference sequence NM_033178.4. This fusion has not been reported before [5], [12]–[14]. *CIC* fusions have been reported at nt 4552, 4579, 4740, 4750 [12]–[14], [16] and for *DUX4* at nt 1071, 1078, and 1145 of the reference sequence with accession number NM_033178.4 [5], [12]–[14].

**Figure 4:**
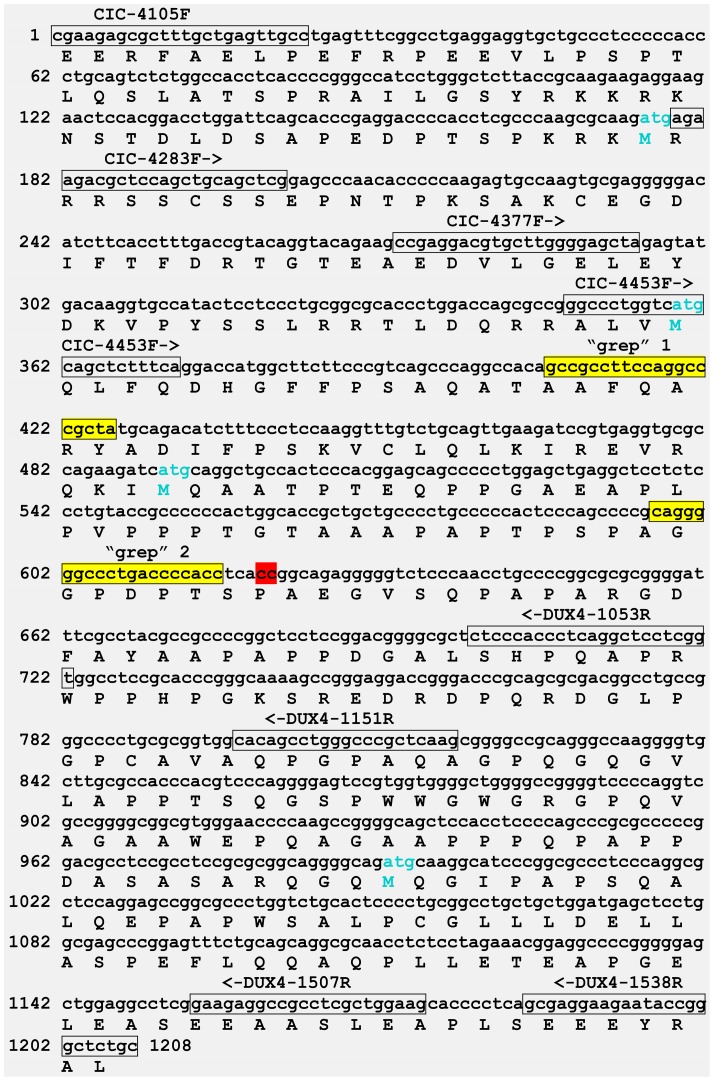
A putative 1208-DUX4 fusion transcript which would have been amplified using the the forward CIC-4105F and reverse DUX4-1538R primers. All the primers used in the study are denoting the primers sequences (in box) together with orientation (arrows). The search sequences “GCCGCCTTCCAGGCCCGCTA” (“grep” 1) and “CAGGGGGCCCTGACCCCACC” (“grep” 2) used as search terms in the “grep” command-line utility are colored yellow and in box. The fusion point between CIC and DUX4 is in red. The part of the protein coded by this CIC-DUX4 fusion transcript fragment is shown under the nucleotide sequence. The nucleotide sequence has been deposited in the GenBank with accession number KJ670706.

An explanation for the failure of the initial PCR is that the target *CIC-DUX4* chimeric sequence between CIC-4105F/DUX4-1538R primers was 1208 bp long with 70% CG content (Figure 4). The primer combinations CIC-4377F/DUX4-1151R and CIC-4453F/DUX4-1053R together with a PrimeSTAR GXL DNA polymerase, suitable for GC-rich templates, amplified fragments 546 bp long with 70% CG content and 374 bp long with 69% CG content, respectively (Figures 3B and 4). Sanger sequencing verified that they were *CIC-DUX4* fusion transcripts which had the same fusion point found with the “grep” command-line utility.

Current knowledge about the *CIC-DUX4* fusion holds that in the encoding protein CIC is mostly preserved and retains its HMG-box domain fusion, while DUX4 has lost most of its sequence, including its two DNA-binding homeodomains [5], [11], [12], [14]. As a consequence of the fusion the transcriptional activity of CIC is enhanced, suggesting an abnormal regulation of downstream targets [5]. CIC–DUX4 directly binds the ERM promoter by recognizing a novel target sequence and significantly up-regulates its expression [5]. Mashado et al [16], on the other hand, described an undifferentiated small round cell sarcoma in which *CIC-DUX4* coded for a putative truncated CIC protein. In that case, the last 104 amino acid residues of CIC protein were deleted and *DUX4* contributed a triplet followed by a stop codon. It is not known whether this truncated CIC protein would have resulted in an enhanced transcriptional activity of CIC.

In conclusion, our study showed that the three fusion-finder programs FusionMap [19], Fusion Finder [20], and ChimeraScan [18] generated a plethora of fusion transcripts but not the biologically important and cancer-specific fusion gene, the *CIC-DUX4* chimeric transcript. It was necessary to use the “grep” command-line utility to sift out the latter from the many data produced by the automated algoritms. Cytogenetic, FISH, and clinico-pathologic tumor features hinted at the presence of the said fusion, but it was eventually found only after the manual “grep”-function had been used.

## Supporting Information

Table S1 Fusion transcripts detected using FusionMap.
(XLSX)

Table S2 Fusion transcripts detected using FusionFinder.
(XLSX)

Table S3 Fusion transcripts detected using ChimeraScan.
(XLSX)
